# Feasibility and Efficacy of the “FUNPALs Playgroup” Intervention to Improve Toddler Dietary and Activity Behaviors: A Pilot Randomized Controlled Trial

**DOI:** 10.3390/ijerph18157828

**Published:** 2021-07-23

**Authors:** Aliye B. Cepni, Ashley Taylor, Christine Crumbley, Debbe Thompson, Nancy E. Moran, Norma Olvera, Daniel P. O’Connor, Katherine R. Arlinghaus, Craig A. Johnston, Tracey A. Ledoux

**Affiliations:** 1 Department of Health and Human Performance, University of Houston, Houston, TX 77204, USA; abcepni@uh.edu (A.B.C.); christine.crumbley@gmail.com (C.C.); dpoconno@central.uh.edu (D.P.O.); cajohn25@central.uh.edu (C.A.J.); 2 Department of Psychological, Health and Learning Sciences, University of Houston, Houston, TX 77204, USA; aataylo4@central.uh.edu; 3 USDA/ARS Children’s Nutrition Research Center and Department of Pediatrics, Baylor College of Medicine, Houston, TX 77030, USA; dit@bcm.edu (D.T.); Nancy.Moran@bcm.edu (N.E.M.); 4 Department of Psychological, Latino Health Disparities Lab, University of Houston, Houston, TX 77204, USA; NOlvera@central.uh.edu; 5 Division of Epidemiology and Community Health, University of Minnesota School of Public Health, Minneapolis, MN 55455, USA; karlingh@umn.edu

**Keywords:** playgroup, early childhood, obesity prevention, diet, activity

## Abstract

This study evaluated the feasibility and effects of the Families Understanding Nutrition and Physically Active Lifestyles (FUNPALs) Playgroup on toddler (12–36-month-old) diet and activity behaviors. Parent–toddler dyads were recruited from disadvantaged communities and randomly assigned to receive 10-weekly sessions of the FUNPALs Playgroup (*n* = 24) or dose-matched health education control group (*n* = 26). FUNPALs Playgroups involved physical and snack activities, delivery of health information, and positive parenting coaching. The control group involved group health education for parents only. Process outcomes (e.g., retention rate, fidelity) and focus groups determined feasibility and perceived effects. To evaluate preliminary effects, validated measures of toddler diet (food frequency questionnaire and a carotenoid biomarker), physical activity (PA; accelerometers), general and feeding parenting (self-report surveys), and home environment (phone interview) were collected pre and post. The sample comprised parents (84% female) who self-identified as Hispanic/Latino (38%) and/or African American (32%). Retention was high (78%). Parents from both groups enjoyed the program and perceived improvements in their children’s health behaviors. Objective measures demonstrated improvement with large effects (η^2^ = 0.29) in toddler diet (*p* < 0.001) but not PA (*p* = 0.099). In conclusion, the FUNPALs Playgroup is feasible and may improve toddler eating behaviors.

## 1. Introduction

Obesity is pervasive, difficult to treat, elevates risk for life-threatening chronic diseases [1], and disproportionately impacts low-income, ethnic minority populations [2]. Obesity-related behavior patterns and preferences, including poor diet quality, physical inactivity, screen media exposure, and inadequate sleep emerge in early childhood [3] and track through life [4,5]. In response, the National Academy of Medicine and the American Academy of Pediatrics recommend that national policies to prevent obesity not only focus on older children and adults but also be directed toward early childhood [6]. Toddlerhood (12–36 months) is a critical period to establish optimal health behaviors because it is a time in life when habits are developing, it is highly modifiable, and almost entirely reliant on external environmental conditions [7].

Healthy diets rich in FV and low in added sugars and saturated and trans fat reduces risks for various chronic diseases and obesity [8]. Further, the Centers for Disease Control and Prevention recommends that children engage in 60 min of daily moderate to vigorous physical activities (MVPA) and reduce sedentary activity for children’s healthy physical, psychosocial, and cognitive development [9]. The American Academy of Pediatrics recommends children of all ages receive adequate amounts of quality sleep because longer sleep duration is associated with better body composition, emotional regulation, and growth among young children [10]. Therefore, in this study, we aimed to promote public health recommendations for diet (i.e., consumption of FV, trans and saturated fat, sugar, and SSB), sleep, MVPA, and sedentary activity among toddlers.

Social Cognitive Theory (SCT) posits that health behaviors are part of a dynamic interplay between individual and environmental factors [11]. Thus, the social and physical aspects of the home environment may be important targets for obesity prevention programs [12]. Despite the significance of the home environment and parenting on child eating and activity behaviors, most early childhood obesity prevention interventions are delivered to children and do not promote changes within the family home environment [13]. Family Systems Theory (FST) suggests that changes within the family system or home environment may be met with resistance because change is thought to cause significant anxiety among system members [14]. Even positive changes within the family system (e.g., removal of sugar-sweetened beverages from the home) may disrupt homeostasis, causing conflict as children attempt to reinstate homeostatic balance with tantrums, food refusal, emotional outbursts, and aggression. For instance, one survey of providers reported parent–child conflict was a significant barrier to effective weight management treatment [15]. FST suggests overcoming this challenge by supporting families’ skills for problem-solving within relationships [14]. Therefore, programs that instruct parents on nutrition and activity recommendations for children without teaching positive parenting skills may be unsuccessful because parents may require more skills to manage their children’s resistance to changes [16]. In this study, we aimed to promote toddlers’ health behaviors by helping parents make positive changes to the home environment (based on SCT) and by helping parents adopt positive parenting skills to manage their child’s resistance to changes (based on FST).

Parent engagement is essential to effective childhood obesity prevention programs [17], but it is challenging, particularly among low-income parents [18]. For example, only 44% of eligible families participate in the United States’s largest federal nutrition education and supplemental food program for 1–4-year-old children (i.e., WIC) [19]. Federal nutrition programs are underutilized [19,20], but profit-based family fitness playgroups are highly successful [21]. In the US, the children’s fitness industry offers fitness and music playgroups to families with young children (e.g., Little Gym^®^, Gymboree^®^), but access varies by income and community [22]. 

Playgroups are organized parent–child groups that meet regularly for social, physical, and educational play [23]. The benefits of playgroups for children include enhanced parent–child attachment, learning competence, social–emotional development, and self-regulation [24]. The benefits of playgroups for parents include reduced stress and isolation, and increased parental wellbeing, sense of belonging, and opportunities to build social capital [25,26]. Playgroups also have the capacity to facilitate parent learning through in vivo coaching, which encourages parents to adopt positive parenting skills via direct instruction, demonstration, observation, and real-time feedback to parents on their approach [27,28]. Given the numerous benefits of playgroups, some countries, such as Australia, Indonesia, and New Zealand have invested in making playgroups available to all families with young children regardless of income and geographic location through an organized network of government-funded associations [29]. Most playgroups in Australia emphasize play for the socio-emotional and educational benefits, but there is evidence that playgroups are a feasible delivery format for health-promotion content [30]. The American Academy of Pediatrics recommends children spend ample time in unstructured and guided playtime with caregivers every day, and playgroup settings may be viable options to simultaneously meet play recommendations and promote health behavior development [31].

To our knowledge, there are no evidence-based childhood obesity prevention playgroups for families with toddler-age children in the US. Therefore, the primary aim of this randomized controlled pilot trial (RCT) was to test the feasibility and acceptability of the Families Understanding Nutrition and Physically Active Lifestyles (FUNPALs) Playgroup in the US. Our secondary aim was to generate preliminary data on the efficacy of FUNPALs Playgroup on toddler health behaviors. We hypothesized that compared to those in the control group, toddlers in the FUNPALs Playgroup would have healthier home environments, diets, and objectively measured activity habits (moderate to vigorous physical activity (MVPA), sedentary activity, and sleep); and parents of toddlers in the FUNPALs Playgroup will have healthier feeding practices. The overarching goal of this pilot study was to inform a fully powered RCT.

## 2. Materials and Methods

### 2.1. Research Design

**Figure 1:**
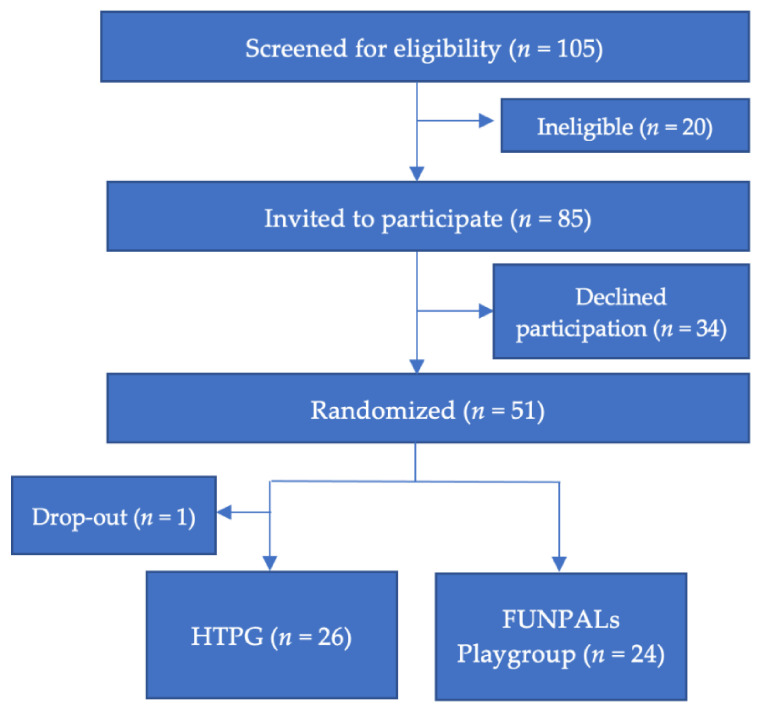
CONSORT diagram illustrating the flow of participants through the study.

The study was a 2-arm, mixed-methods, randomized controlled pilot trial (RCT) with participants randomly assigned to the FUNPALs Playgroup (treatment group) or the dose-matched Healthy Toddler Parent Group (HTPG; control group). At baseline and immediate post, families completed quantitative measures of outcomes. Immediately post-intervention, families completed focus group interviews and a satisfaction survey. Process evaluation variables were tracked throughout the study. The Consolidated Standard of Reporting Trials (CONSORT) guidelines for pilot and/or feasibility studies were followed to report the current study findings [32]. The overall study design is presented in a CONSORT diagram in Figure 1. All parents provided written informed consent for themselves and their toddler-age child to participate. The study was conducted according to the guidelines of the Declaration of Helsinki and approved by the Institutional Review Board of the University of Houston (no. 1076/9 July 2018).

### 2.2. Recruitment and Participants

As we wanted to test the feasibility of the intervention and study design in low-income and ethnically diverse parents with toddler-age children (12–36 months), we attempted to recruit participants mainly from the Greater Third Ward neighborhood in Houston, Texas, where >80% of the residents are identified as either African American (AA) or Hispanic and 35%–54% considered low income (<25,000 USD per household) [33]. To ensure that our recruitment materials reflected intervention and treatment control programs, the materials stated that we were conducting a family wellness program. Recruitment strategies included listing study information on social media advertisements (e.g., Facebook), flyers distribution around community centers (e.g., daycare centers, apartment complexes), and in-person recruitment by research staff at community centers (e.g., children’s museum, libraries, churches, health care provider offices) between September 2018 and April 2019. Parents who were interested in participating contacted research staff by phone or email. Screening was completed by phone or email before potential participants were invited to a data collection and enrollment session.

To be included, parents had to be (1) the legal guardian of a toddler, (2) at least 18 years old, (3) able to make food decisions in the home, (4) fluent in English, and (5) have access to a phone at home; toddlers must be (1) between 12 and 36 months of age and (2) able to walk to attend activities. Only one parent per toddler was included in the study and completed the measurements. If the parent has more than one toddler-age child, they select the child they wish to enroll in this study. Parents and toddlers with severe health problems (e.g., reliance on feeding tube, food allergy, severe asthma) that would prevent them from fully engaging in the program and activities were excluded.

### 2.3. Sample Size and Randomization

Viechtbauer’s best practices for estimating pilot study sample size were utilized [34]. Given that a goal of this study was to ascertain perceived barriers and facilitators of recruitment and retention, we wanted a sample size large enough to replicate the average attrition rate observed in parent-focused interventions. The average attrition rate among minority participants in parent interventions often exceeds 30% [35]. Therefore, a sample of 50 parent–toddler dyads allowed us to estimate an attrition rate of 30% ± 6.5 with 95% confidence [34].

Participants were randomized to one of two groups (in a 1:1 ratio). A researcher not involved in data collection generated computer-based block randomization sequences (random block sizes of 2–6) and prepared concealed envelopes revealed at baseline data collection event after measures were completed.

### 2.4. Procedure

Recruitment and data collection were completed in two waves (Fall 2018 and Spring 2019). At the beginning of each wave, 1–2 weeks before the intervention start date, participants attended 90 min group data collection sessions at a community health and fitness center (The Houston Texans YMCA) where they completed the consent form and then filled out baseline measures, received the accelerometers, and scheduled a time for the home environment phone survey. After data was collected, participants were randomly assigned to the experimental or control groups. During data collection sessions immediate post, 1–2 weeks after the 10-week intervention, participants completed follow-up measures, returned accelerometers, and participated in a focus group. Home visits were arranged for participants who were unable to attend any of the scheduled data collection sessions. All data collection sessions were conducted by trained research staff. Participants received a 30 USD store gift card at the end of each data collection session, a 20 USD store gift card for completing the home environment phone survey, and some earned an extra 10 USD gift card (up to a maximum of 30 USD) as an incentive for each participant they referred to the study.

### 2.5. Theoretical Framework

The Intervention Mapping (IM) framework was used to develop the FUNPALs Playgroup curriculum [36]. The FUNPALs Playgroup curriculum was developed based on research, theory, target audience feedback, and public health recommendations [8,9,10]. Based on Social Cognitive Theory, the FUNPALs Playgroup focused on experiential learning strategies to facilitate positive change in the social and physical aspects of the toddler’s home environment [11]. The FUNPALs Playgroup was based on Family System Theory in that parents were taught how to overcome anticipated toddler resistance to home environment changes with general positive parenting strategies. Self-determination theory suggests motivation to change is related to the degree to which the basic psychological needs of autonomy, competence, and relatedness are satisfied [37]. The FUNPALs Playgroup facilitators were trained to provide an environment where participants felt supported in their autonomy, a growing sense of competence over the target behaviors (structured feeding and creating positive parent–child interactions), and a sense of belonging for learning the target behaviors. A diverse group of key informants (i.e., parents of toddlers) and community stakeholders (i.e., early childhood education center directors, community center directors, YMCA program directors) selected from Third Ward neighborhood were engaged to help the research team design, modify, and implement the curriculum to ensure cultural relevance and appeal. The FUNPALs Playgroup weekly lesson topics are presented in Table 1.

### 2.6. Experimental Group

The FUNPALs Playgroup sessions were delivered to participants and their family members for 10 weeks on Saturday mornings. The FUNPALs Playgroup participants met in a large community nutrition education center located on the ground floor of a health services building on the University of Houston campus. The building was easily accessible via commuter rail and bus and had free parking out front. Two community health workers were trained to lead the 90 min playgroup sessions according to the lesson plans, which included the following: (1) an opening welcome song that included each child’s name to enhance a sense of belonging, (2) five short moderate to vigorous physical activities (MVPAs), (3) in vivo parent coaching breaks, (4) a snack preparation activity, in which participants were provided with ingredients to assemble an age-appropriate, healthy snack (e.g., banana, strawberry, nut butter, whole-grain tortilla wrap) with child-safe tools, and bibs, (5) a relaxing yoga activity, and (6) good-bye song. During in vivo coaching breaks, toddlers were given quiet sensory toys while the facilitators gave the parents information about nutrition, parenting, and activity (See Table 1—FUNPALs Playgroup Curriculum Topics) and then gave parents feedback as they practiced the skill with their child. At the end of each playgroup, parents received handouts: one for the snack recipe prepared during playgroup with related price information and a map of stores where food ingredients could be purchased, and another for a summary of the nutrition, PA, and parenting topics discussed in playgroup.

To improve retention, participants were reminded via emails and text messages each week of the upcoming playgroup session [17]. Further, a password-protected website and a private Facebook group were created for parents where they could access additional resources, including videos of cooking demonstrations for each week’s featured recipe, pictures taken from each playgroup, educational posts related to each week’s lesson, and the handouts [38].

### 2.7. Control Group

Participants in the HTPG participated in 10 weekly 90 min health education sessions for parents at a community health and fitness center (i.e., YMCA). HTPG sessions were modeled after the WIC program, which is the largest federal nutrition education program for families with toddler-age children in the United States [19]. The HTPG was led by a trained facilitator, who reviewed the same nutrition and physical activity lessons as the experimental group and encouraged discussion among the participants on the topics. In contrast to the experimental group, control group parents did not receive lessons or in vivo coaching on parenting, did not participate in a playgroup setting with their child, and did not engage in experiential learning activities such as snack preparation and guided PA. The HTPG received a handout that contained only a summary of nutrition and PA information from the session. This handout did not include the recipe, food store maps, or parenting information that was presented to the FUNPALs participants. Free childcare service was provided for study children and siblings during the class. The HTPG also were asked to complete a satisfaction survey at the end of each session.

### 2.8. Process Evaluation Outcome Measurements

#### 2.8.1. Facilitators and Barriers of Recruitment

At screening, participants provided an open response regarding the reason(s) they wanted to sign up for the study and the reasons they were not interested in participating. 

#### 2.8.2. Demographics

Demographic data were self-reported via baseline surveys and included participating parents’ and child’s age, sex; parents’ education level; marital status; annual household income; and race and ethnicity.

#### 2.8.3. Participants Engagement

Parents completed the Interest/Enjoyment subscale of the Intrinsic Motivation Inventory at the completion of the study [39,40]. Parents reported the extent to which they found the groups fun, enjoyable, boring (reverse scored), and interesting on a 7-point Likert scale (1 = very untrue, 7 = very true).

#### 2.8.4. Facilitators and Barriers of Retention and Perceived Impact

Retention is defined as the frequency of the participants who attended baseline and post-testing, which was utilized to assess feasibility of this study. Focus groups allow investigators to openly explore a phenomenon from the perspective of the target audience [41]. Parents were invited to participate in focus groups after the interventions were complete. Focus groups were conducted with parents from each intervention group separately. Overall, 13 focus groups were offered for the FUNPALs Playgroup (9 groups) and HTPG group (4 groups). One to five attendees participated in each focus group. Doctoral-level qualitative researchers facilitated the focus groups using a semi-structured interview guide. A trained research assistant recorded notes during focus groups. The focus groups lasted 45–60 min each, and interviews were audio-recorded. Sample questions included:

- “What motivated you to come to playgroup/class every week?”;
- “What barriers did you face coming to playgroup/class every week?”;
- “To what extent did the playgroup/class help your family make changes in nutrition/physical activity/parenting?”

The interview guides are presented as supplementary material (Supplementary S1 and Supplementary S2).

#### 2.8.5. Fidelity

Trained research staff observed every playgroup session and rated facilitators on a 4-point Likert scale (4 = a lot, 1= not at all) on the extent to which the facilitator created a playgroup environment that encouraged autonomous motivation. Observers also recorded attendance and duration of each portion of the lesson plan.

### 2.9. Preliminary Effect Outcome Measurements

#### 2.9.1. Dietary Intake of Children

The Kids Bites Food Frequency Questionnaire (27 items), a modified version of a validated beverage and snack questionnaire (BSQ), asked parents to report how often their child consumed specific fruits, vegetables, snack foods, and beverages during the past week [42]. The Kids Bite FFQ was developed and demonstrated construct validity and internal reliability among samples of toddlers and preschoolers [43]. Child fruit and vegetable (FV) intake was further assessed by non-invasive measurement of skin carotenoids by pressure-mediated reflection spectroscopy (Veggie Meter, Longevity Link Corp., Salt Lake City, UT, USA). This is a biomarker of carotenoid status, carotenoid intake, and FV intake [44]. For this measure, toddlers placed their right index finger on a lens, which was then pressed down using a pressure lever. The device provides a linear score that represents skin carotenoid concentrations, which can range from 0 to 800. A higher score indicates higher concentration of carotenoids in the skin. The Veggie Meter provides scan results using one of two features. The triplicate reading feature involves taking three rapid scans for 90 s. The single reading feature involves taking one reading for 10 s. For toddlers who were able to hold their fingers in the scanner for 90 s, the triplicate feature was used. For toddlers who were not able to hold their fingers in the scanner for 90 s, two separate readings were collected. If the values from the two separate readings were more than 33 units apart (represents 1 SD from reference population data), a third reading was collected, and the two closest values were averaged.

#### 2.9.2. Children’s Activity and Sleep

Parents were instructed to have their children wear the accelerometer (GT3X + Actigraph, Pensacola, FL, USA) on the right hip 24 h/day for 8 consecutive days, which allowed the child to adapt to wearing the device and to achieve the study goal of 4 valid wear days per child [45]. Previous research has suggested that 4 days of wear time can provide information on usual activity patterns for toddlers [46]. Parents were instructed to fill out activity logs to track their child’s bedtimes, wake-up times, nap times, bath times, and other periods where the accelerometer may have been removed (e.g., the child went swimming, the child cried about wearing the belt, etc.). Data were downloaded using 60 s epochs and processed using ActiLife 6 software (Actigraph, Pensacola, FL, USA) provided by the manufacturer. Sleep periods were marked in the ActiLife program based on the activity logs, then analyzed using the program’s Sadeh algorithm. If there was a period of activity 5 or more consecutive minutes during the sleep hours, the active period was removed from the sleep period and considered awake time. A valid day was defined as ≥600 min wear time per day, as previous studies reported [47]. Sleep period and 40 min of consecutive zero counts were considered as non-wear time and excluded from the wear time analysis. Mean values of sedentary time and MVPA time per child were calculated.

#### 2.9.3. Feeding Practices

The Structure and Control Parent Feeding Questionnaire was previously developed for assessment of “Structure” and “Control” in feeding practices [48]. Only the 22-item Structure subscale was utilized in this study to determine covert parent feeding practices, which include limiting exposure to unhealthy foods (11 items; Cronbach’s alpha = 0.79) and establishing mealtime routines (11 items; Cronbach’s alpha = 0.75). The Limit Setting subscale of structure feeding measures the degree to which parents discourage energy-dense foods consumption. The Consistent Feeding Routines subscale of structure feeding measures consistency in mealtime habits and timing of meals. Participants graded the items on a 5-point Likert scale: 0 = never and 4 = always. Strong use of structure in feeding children is demonstrated by high scores. In the current study, the questionnaire indicated good reliability (Cronbach’s alpha = 0.82).

#### 2.9.4. Home Environment

The physical (availability and accessibility of resources) and social (norms and policies) aspects of the home environment related to food, physical activity, sleep, and screen media were assessed by using validated items from the Healthy Homes Survey [49], the Home Food Inventory [50], and the Sleep Environment Questionnaire [51], and some items were developed for this study (e.g., those regarding availability of mobile devices, parent screen media and sleep modeling, and family policies around sleep). All survey items except the Home Food Inventory were completed via pencil and paper. The Home Food Inventory items were completed via a 20–30 min phone interview. When it was not possible to schedule a phone call, the hard copy of the interview was delivered to the participants via email or by hand for completion (*n* = 5). In calculation of the home environment composite score, each item was standardized using z-score. Leptogenic constructs (e.g., number of fruits available in the home) were positively scored, and the obesogenic constructs (e.g., availability of screens) were reverse scored. All items were summed to produce one composite score in which higher scores represent a healthier (i.e., leptogenic) home environment and low scores represent an unhealthy (i.e., obesogenic) environment. The detail of the home environment composite score calculation has been published previously [52]. The instrument used in this study for home environment assessment is available by request.

#### 2.9.5. Anthropometrics

Trained research staff collected child height and weight using standardized procedures [53]. Toddler’s weight was measured in duplicate to the nearest 0.1 kg using a digital scale. Heights were measured in duplicate to the nearest 0.1 cm with a stadiometer. Child BMI-for-age percentiles were calculated using World Health Organization age and sex-specific growth curves [54]. BMI-for-age percentile has been found as an appropriate indicator of growth among children, including children under 2 years old [55]. Parental body mass index (BMI) was assessed using the Quetelet index: *weight (kg)/height (m^2^)* from self-reported data at baseline [56].

### 2.10. Statistical Analysis

All analyses were performed using SPSS version 23 (IBM, Armonk, NY, USA). Missing data were treated with k-nearest neighbor (NN) imputation, which is found feasible to produce real-life data in studies with a small sample size [57]. The value of “k” was set to 3 to minimize imputation error and protect the data structure [58]. Parental BMI has been found associated with children’s health behavior development [59]. Further, age of children and received intervention dose can influence success of family-based childhood obesity prevention programs [60,61]. Therefore, nearest neighbors were determined by computing the distance between the recipient (missing case) and all other subjects (candidate donors) based on the three variables mentioned above. Then, the values from three donors with the minimum calculated distance were averaged to complete the recipient’s missing data. Using intent-to-treat analysis, we performed 2 (group) by 2 (time) repeated measures ANOVA to examine differences between the intervention and control group from baseline to post-intervention on diet, MVPA, sleep, home environment, and parent feeding practices. The results presented were differences in the means and 95% confidence intervals, and the statistical significance of the interaction effect between group and time was determined using *p* ≤ 0.05.

Identification of perceived effects of the intervention was assessed via focus groups. Focus Group analyses proceeded using the Rapid Identification of Themes from Audio Recordings (RITA) process [62]. The RITA procedure involved (1) listening to the audio recordings several times using a constant comparison method to identify major themes, (2) constructing a codebook defining the major themes, (3) breaking audio recordings into 3 min segments, and (4) listening to the audio recording segments while quantifying the number of themes discussed by participants during each segment. To increase the validity and reliability of the coding process, all coders were trained to ensure that they understand the codebook definitions and the procedures for coding by time segments [62]. All interviews were coded by two coders using a constant comparison approach. Coders met regularly throughout the coding process, and the lead investigator helped coders resolve disputes as needed. Further, our sample size allowed us to achieve thematic saturation, the point at which all themes have been identified from qualitative data [63], a minimum of 9 participants were required [64].

## 3. Results

### 3.1. Sample Characteristics

Participants’ baseline characteristics are presented in Table 2. There were no significant differences in these characteristics among the two groups (*p* > 0.05), except toddler snack intake. Toddlers in HTPG had higher snack consumption compared to FUNPALs Playgroup at baseline (*p* = 0.028). The mean age was 32 years for the parents and 23 months for the toddlers (Table 2). Most of the participants were mothers (84%), married/cohabitating with partners (60%), and had a college degree or greater (62%). Most participants self-identified as either Hispanic/Latino (38%) or African American (32%). Most participants had an average annual household income of less than 50,000 USD (56%). The characteristics of the sample were consistent with the community where the sample was recruited [33]. The mean toddler BMI-for-age percentile was 76.2, which is considered a healthy weight. The mean parental BMI was 28.4 kg/m^2^, which is considered overweight.

### 3.2. Recruitment and Retention: Facilitators and Barriers

A total of 105 families were screened, 85 eligible families were invited to participate in the study, and 51 families completed baseline assessments and were randomized to the study arms within 4 months. Thus, the recruitment goal was met. Among those who enrolled in the study, the most popular recruitment methods were Facebook (*n* = 21), a referral from a friend (*n* = 6), a pediatrician office (*n* = 6), and the local children’s museum (*n* = 5). The most common parent-reported reasons for joining the study were the opportunity for their child to socialize with other children (*n* = 14), socialize with other parents (*n* = 11), spend quality time with their child (*n* = 9), and learn about parenting (*n* = 9). The only barrier reported for joining the study was having a schedule conflict (*n* = 5). The response rate to the pre- and post-questionnaires was 78% among the total participants. 

Among participants randomized to the FUNPALs Playgroup, 79.2% completed measures at baseline and post-intervention. Participants attended an average of 5.5/10 sessions (SD 3.2). No participants withdrew from the FUNPALs Playgroup, but three participants (12.5%) never attended the playgroup. Focus groups revealed there were two main barriers to retention in the FUNPALs Playgroup: personal issues and perceived chaotic playgroup environment. Participants reported missing all or some playgroup sessions due to personal issues such as work schedule conflicts, custody arrangements, and illness. Some participants perceived the FUNPALs Playgroup to be chaotic, with some children, particularly older siblings of enrolled toddlers, dominating some of the activities at the expense of the youngest toddlers in the study. Parents reported during focus groups that they attended some or all the playgroups because (1) children enjoyed the playgroup, (2) children had the opportunity to socialize with other children, and (3) families had the opportunity for fun quality time together.

Among HTPG participants, 76.9% completed measures at baseline and post-intervention. Participants attended an average of 4.0/10 sessions (SD 3.4), and this was not significantly different from FUNPALs Playgroup attendance. One HTPG participant withdrew because she was uncomfortable being part of a research study. Six HTPG participants (23%) never attended the class. Focus groups revealed that parents missed all or some classes due to personal issues (e.g., illness, schedule conflicts) or lack of interest in the topics covered. Parents reported attending all or some of the HTPG classes for the social aspects. They enjoyed discussions with other parents and the facilitator.

### 3.3. Perceived Impact

*FUNPALS Playgroup*. Regarding nutrition, parents reported during focus groups that participation in the FUNPALs Playgroup led to new ideas for meals and snacks, healthier food choices, and increased awareness of age-appropriate dietary needs for toddlers. Regarding physical activity, parents reported that participation in the FUNPALs Playgroup led to increased visits to the park and knowledge of activity recommendations for toddlers. Regarding positive parenting skills, parents reported that participation in the FUNPALs Playgroup led to increases in positive food parenting practices, general positive parenting skills, and increased communication with their children.

*HTPG*. Regarding nutrition, parents reported that participation in the HTPG led to healthier food choices and new ideas for meals and snacks. Regarding physical activity, parents reported that the HTPG led to their family trying new physical activities at home. Regarding positive parenting, parents reported that the HTPG either promoted more interaction with their child or had no effect on their parenting.

### 3.4. Fidelity

*FUNPALs Playgroup*. The fidelity checklists revealed that facilitators covered 100% of the required content and created an environment that supported autonomous motivation (per Social Determination Theory), which is one where participants felt they belonged, where they felt competent in performing target behaviors, and where their autonomy is supported. On a 4-point Likert scale, trained observers rated the extent to which parents/families appeared to be engaged (3.95), to enjoy (3.95), to relate to other families (3.26), to feel comfortable with the facilitators (3.74), and to have autonomy (3.83) as very high. On a 4-point Likert scale, trained observers rated the extent to which facilitators were engaging (3.89), nonjudgmental (4.00), and supportive (4.00) as very high. Observers also reported that the degree to which the playgroups were chaotic as being “not much.” On an engagement survey of intrinsic motivation, FUNPALs Playgroup parents reported it was “true” (5.7 out of 7 = very true) that they found the program enjoyable, fun, and interesting.

*HTPG.* The fidelity checklists revealed facilitators covered 100% of the required content. On a 4-point Likert scale, trained observers rated the extent to which parents appeared to be engaged (3.89), to enjoy (3.94), to relate to other parents (3.83), and to feel comfortable with the facilitators (4.00) as very high. On a 4-point Likert scale, trained observers rated the extent to which facilitators were engaging (3.94), nonjudgmental (4.00), and supportive (4.00) as very high. On an engagement survey of intrinsic motivation, HTPG parents reported it was “true” (5.4 out of 7 = very true) that they found the program enjoyable, fun, and interesting.

### 3.5. Preliminary Effects

Table 3 shows the changes in toddler outcomes. A time by group interaction effect demonstrated that toddlers in the FUNPALs Playgroup significantly reduced their SSB intake by 1–2 servings per week compared to toddlers in the HTPG, who increased SSB intake (*p* < 0.001, *η*^2^ = 0.29). There was a main effect of time on toddler skin carotenoid scores (SCS) wherein both groups experienced increases from pre- to post-intervention (*p* = 0.014, *η*^2^ = 0.12). The SCS were correlated with FV consumption measured via FFQ after controlling for BMI percentile (*r* = 0.31, *p* = 0.03). There were no other significant interactions or main effects on diet, activity, home environment, or parenting variables. However, among FUNPALs Playgroup participants, there were non-significant changes in the desired direction on snack consumption, FV consumption (FFQ measure), sleep, and positive parent feeding behaviors.

## 4. Discussion

There is a need for family-based obesity prevention programs that engage low-income ethnic minority families [17]. Playgroups are effectively and widely utilized in some countries to promote early childhood development [65]. In the US, there is a profitable playgroup industry suggesting interest and demand, but participation in these playgroups is costly and likely not feasible or accessible to low-income families. To address this issue, this study examined the feasibility, acceptability, and perceived effects of a playgroup program, FUNPALs Playgroup, for delivering obesity prevention strategies to low-income families. We found that our study design was feasible to deliver, families found the FUNPALs Playgroup highly acceptable, and parents reported participation in the FUNPALs Playgroup led to positive changes in their parenting skills and in their toddlers’ diet and activity. Our secondary aim was to explore the effects of the FUNPALs Playgroup on toddler diet, toddler activity, the home environment, and parenting skills. Results suggest the FUNPALs Playgroup may have a large positive effect on certain aspects of toddlers’ diet but may have smaller, if any, effects on toddlers’ activity behaviors and parental feeding practices.

In this pilot study, we recruited a sufficient number of low-income, ethnically diverse parents to detect the feasibility of the intervention. A multi-faceted recruitment strategy and appeal of the playgroup format were critical to successful recruitment. Others have also found recruiting through multiple avenues on multiple occasions to be successful [17]. A growing body of literature suggests that social media platforms, particularly Facebook, are valuable and cost-effective recruitment tools for health behavior change interventions [66,67]. Similarly, in this study, Facebook was identified as the most efficient recruitment tool. Consistent with existing research, many families reported joining the study because of the opportunity to socialize with other families and to spend fun quality time with their children [17,68]. It is possible there will be greater success in obesity prevention program enrollment if recruitment is conducted via a variety of methods, including social media, and if programs offer families fun opportunities to socialize with other families. 

The overall retention rate for the current study (78%) was notably high compared with existing childhood obesity intervention studies, which reported retention rates ranging from 27% to 73% among low-income and ethnic minority populations. [69,70]. Evidence-based strategies to increase parent retention were utilized. For example, we provided reminder text messages in the lead-up to sessions. Additionally, there was no need for childcare in either group as control group families were provided a free childcare service, and the FUNPALs Playgroup included the children [17,70,71]. In focus groups, parents from the control group reported that the opportunity to socialize with other parents was the primary motivation to attend classes each week. However, FUNPALs Playgroup sessions attendance rate (5.5/10 sessions) was non-significantly higher than the dose-matched control group (4.0/10 sessions). Previous research has shown that program attendance is improved by child engagement, which could explain the increased participation in the FUNPALs Playgroup compared to the control group that did not include the toddlers [17]. Further, the FUNPALs Playgroup parents reported that they attended sessions because their child enjoyed the program and because parents were able to spend quality time with their child [17]. Moreover, fidelity observations confirmed that facilitators provided an environment that included the necessary conditions for motivation to engage in group activities per Self Determination Theory, and this may have enhanced retention. Scheduling conflict was the primary barrier to retention in both study arms, which is similar to existing literature [68,70]. To overcome this barrier, providing make-up sessions in future studies could serve as a mitigation strategy. In the FUNPALs Playgroup, an additional barrier was the sometimes chaotic playgroup environment, in which older siblings and study children dominated some of the activities, making it difficult for the younger participants to engage. In future FUNPALs Playgroups, it may be better to stratify the playgroups by age group and include an extra assistant who can work with older siblings so that parents can focus on the target child. Overall findings suggest that the playgroup design is a unique opportunity to provide entertaining and engaging activities for children, which could improve the compliance of families.

The FUNPALs Playgroup may impact dietary intake outcomes among toddlers. Toddlers in the FUNPALs Playgroup decreased their SSB intake while toddlers in the health education group increased their SSB intake, and this effect was large [72]. Both FUNPALs Playgroup and health education group toddlers increased FV consumption per skin carotenoid scans from pre- to post-intervention, and this effect was large [72]. FV consumption assessed via the food frequency questionnaire did not increase over the course of the intervention, nor did it differ between the groups. This disagreement between the proxy-reported food intakes (i.e., FFQ) and the objective biomarker of FV intake (i.e., skin carotenoid scans) is not surprising, based on the well-known imprecision of dietary recall tools and challenges for single-proxy reporting of pediatric food intake [73]. Skin carotenoid measures have been shown to be highly correlated with carotenoid intake and plasma carotenoid concentrations, which in turn are significantly related to FV intake [74,75]. However, the validity of SCS as a biomarker of total FV intake is dependent on the homogenous incorporation of carotenoid-rich FVs into the diets of the population being studied; therefore, there may be some reduced biomarker sensitivity in small group sizes. These preliminary results are consistent with dietary outcomes of similar playgroups in Australia [65]. Resources (cooking demonstrations, recipes, grocery store maps) were specifically provided to help families extend dietary behaviors practiced in the FUNPALs Playgroup to the home.

The FUNPALs Playgroup did not appear to improve physical activity behaviors among toddlers, although in focus groups, parents reported positive effects on their child’s PA [30]. Differences in findings may be due to differential measures for physical activity. Our study used accelerometry, whereas others have relied on parent-report of child activity. It is also possible that the structured MVPA activities during FUNPALs Playgroup were offset outside of FUNPALs Playgroup sessions. In other words, participants may not have sought out opportunities for physical activity because they knew they would engage in MVPA during FUNPALs. The increase in MVPA among the control group is consistent with that notion, as parents in this group may have felt as though they had to initiate activity outside of class because their child was not receiving structured physical activity in the playgroup. Notably, concomitant with increased child physical activity reported in other studies was increased frequency of taking a child to a place for physical activity [65] and increased frequency of parents playing with their children [30]. During focus groups, parents from the FUNPALs Playgroup reported taking their children to the park more often. In contrast to nutrition, in which parents were provided additional resources to help the promotion of healthy eating behaviors at home, resources related to physical activity were not provided. Integrating additional parent training on how to be active with children outside of the playgroup and providing resources (e.g., community fitness center membership) may enhance physical activity outcomes from FUNPALs Playgroup.

Lastly, we did not find a statistically significant change in parental feeding practices. This is in contrast to Pathirana et al. (2018), who found significant improvement in parental healthy feeding practices via playgroup setting [65]. In our study, parents had high parental feeding practices score at baseline, which may have prevented us from determining the true extent of the treatment effect. However, the direction of parent feeding practices was in a desirable direction, and FUNPALs Playgroup parents reported improvement in their positive parenting practices in focus groups. In this study, we were unable to observe desirable changes in the home environment of toddlers. This lack of effect may indicate that the FUNPALs Playgroup does not adequately address healthy home environment changes. Future implementations of FUNPALs study should emphasize home environment messages and explore methods (e.g., adding more content and/or talking about home environment change messages at the beginning of the program versus the end) by which to motivate parents to change their child’s home environment, such as setting limits to screen-media time, adding routine to bedtime, or being role models for health behaviors.

### Strengths and Limitations

This study may be the first childhood obesity prevention intervention delivered in a playgroup setting to multiethnic groups of parents, providing novel evidence in a field where more primary research is needed [68]. An ethnically diverse group of parents of toddlers and community stakeholders was utilized to inform the development and refinement of the FUNPALs Playgroup curriculum [76]. The use of a mixed-methods design allowed us to interpret our quantitative findings further and explore strategies to optimize the FUNPALs Playgroup. Another strength of this study is that we used objective measures to assess dietary carotenoid intake (biomarker of total FV intake) and activity behaviors of toddlers.

It is important to consider some limitations while interpreting our results. First, this study is a feasibility and pilot trial that was not fully powered and had insufficient sensitivity to evaluate efficacy for primary outcomes. A fully powered RCT study design is needed to confirm our findings. Second, this study included a sample of ethnically diverse lower-income parents, so findings may not be generalizable to the other groups. The RITA procedure analyzing the focus group data has been validated previously [62], but the intercoder reliability for this study has not been tested. However, a subset of interviews was coded by two trained coders, and the third person resolved discrepancies to ensure reliability. Finally, we did not test inter- and intra-rater reliability of the anthropometric measures and utilized self-report surveys to assess the home environment and parenting behaviors, and these types of measures may be subject to bias. Yet, as presented in the method section, the measures demonstrated acceptable reliability and validity.

## 5. Conclusions

The FUNPALs Playgroup study is a novel family-based health promotion intervention for low-income ethnic minority families with toddler-age children. It was rigorously developed by a multidisciplinary team of investigators based on the Intervention Mapping Framework with target audience guidance. The FUNPALs Playgroup is appealing because it is one of the very few family-focused programs for toddlers, whose health behaviors are highly modifiable. Additionally, the FUNPALs Playgroup was fun and engaging for all family members, who are traditionally difficult to engage (i.e., parents from diverse racial, ethnic, and socioeconomic backgrounds). Preliminary and perceived effects suggest the FUNPALs Playgroup may have large positive effects on toddler diet and small-to-moderate effects on PA, if any. Future research should test the efficacy of the FUNPALs Playgroup on the diet and activity behaviors of toddlers. Practice implications of this study are that playgroups may be a vehicle for promoting health behaviors among families with toddlers.

## Figures and Tables

**Table 1:** FUNPALs Playgroup curriculum topics.

Lesson	Parenting Topic	Activity Topic	Nutrition Topic
1	Active listening	MVPA recommendations	Role of parents at mealtimes
2	Imitation play	Types of activities for health	Importance of fruit
3	Descriptive communication	Guided activity	Eat more veggies!
4	Positive reinforcement	Unguided activity	Importance of whole grains
5	Giving your child good directions	Activity in the home	Reducing fat intake
6	Using the choices technique	Sleep	Reducing sugar
7	Delivering consequences	Reducing sedentary activity	Picky eating
8	Using timeouts effectively	Limiting screen time	Meal planning and grocery shopping
9	Dealing with tantrums	Overcoming barriers to activity	Healthy home environment
10	Review	Review	Review

**Table 2:** Characteristics of the sample in total and by group assignment.

	Total (*n* = 50, 100%)	FP (*n* = 24, 48%)	HTPG (*n* = 26, 52%)	*p*
**Parental Characteristics**				
Age (years), mean (SD)	31.7 (5.7)	31.5 (6.1)	31.8 (5.4)	0.842
Female, *n* (%)	42 (84)	19 (79.2)	23 (88.5)	0.199
Race/ethnicity, *n* (%)				0.706
Hispanic/Latino	19 (38)	10 (41.7)	9 (34.6)	
Non-Hispanic White	6 (12)	3 (12.5)	3 (11.5)	
Non-Hispanic African American	16 (32)	8 (33.3)	8 (30.8)	
Asian	7 (14)	2 (8.3)	5 (19.2)	
Other	1 (2)	0 (0)	1 (3.8)	
Marital Status, *n* (%)				0.221
Married or cohabiting	30 (60)	12 (50)	18 (69.2)	
Single	19 (38)	11 (45.8)	8 (30.8)	
Education status, *n* (%)				0.545
High school graduate	5 (10)	3 (12.5)	2 (7.7)	
Some college or technical school	14 (28)	8 (33.3)	6 (23.1)	
College graduate	31 (62)	13 (54.2)	18 (69.2)	
Household annual income, *n* (%)				0.062
<24,999 USD	14 (28)	10 (41.7)	4 (15.4)	
25,000 to 49,999 USD	14 (28)	6 (25)	8 (30.8)	
≥50,000 USD	17 (34)	5 (20.8)	12 (46.2)	
BMI (kg/m^2^), mean (SD)	28.4 (6.4)	27.3 (6.3)	29.4 (6.4)	0.259
**Toddler characteristics**				
Age (months), mean (SD)	22.6 (6.6)	22.3 (6.8)	22.9 (6.5)	0.722
Female, *n* (%)	21 (42)	10 (41.7)	11 (42.3)	0.963
BMI-for-age percentile, mean (SD)	76.2 (25.8)	70.8 (32.2)	80.9 (17.8)	0.191
Toddler outcomes, mean (SD)				
Snack intake	17.9 (4.9)	19.5 (4.8)	16.5 (4.6)	**0.028**
FV intake (FFQ)	32.1 (8.4)	30.9 (7.5)	33.3 (9.2)	0.321
SSB intake	4.5 (2)	5 (2.2)	4 (1.8)	0.083
FV intake (SCS)	253.4 (121.8)	276.9 (126.9)	231.7 (115.2)	0.193
MVPA (min)	26.2 (14.7)	27.1 (16.1)	25.4 (13.5)	0.677
Sleep (min)	612.2 (51.5)	607.7 (52.8)	616.4 (50.9)	0.555

BMI, body mass index; FV, fruits and vegetables; SSB, sugar-sweetened beverage; SCS, skin carotenoid scores.

**Table 3:** Results of repeated measure ANOVA analyses examining changes in parenting behaviors and child health behaviors within and between the study arms from baseline to post-intervention (*n* = 50).

	Adjusted Difference within Groups		Adjusted Difference between Groups
	FUNPALs (T) (*n* = 24)	HTPG (C) (*n* = 26)	T vs. C
**Outcome Variable**	Mean (95% CI)	Mean (95% CI)	Mean (95% CI)
Snack intake	−1.4 (−3.60 to −1.04) *p* = 0.207	0.6 (−1.00 to 2.16) *p* = 0.458	−0.4 (−1.71 to 0.90); *p* = 0.536 G × T; *p* = 0.137
FV intake (FFQ)	1.2 (−2.06 to 4.37) *p* = 0.466	−1.8 (−5.06 to 1.46) *p* = 0.266	−0.3 (−2.56 to 1.91); *p* = 0.772 G × T; *p* = 0.190
SSB intake **	−0.7 (−1.35 to −0.14) *p* = 0.018	1.1 (0.47 to 1.72) *p* = 0.001	0.2 (−0.25 to 0.60); *p* = 0.407 **G × T; *p* < 0.001**
FV intake (SCS) *	70.7 (−1.29 to 142.75) *p* = 0.054	51.6 (−16.8 to 119.98) *p* = 0.133	61.2 (12.79 to 109.51); ***p* = 0.014** G × T; *p* = 0.692
MVPA (min)	−2.9 (−9.07 to 3.30) *p* = 0.344	7.1 (−3.18 to 17.47) *p* = 0.166	2.1 (−3.86 to 8.12); *p* = 0.479 G × T; *p* = 0.099
Sleep (min)	6.9 (−13.24 to 27.00) *p* = 0.486	−18.2 (−39.02 to 2.71) *p* = 0.085	−5.6 (−19.80 to 8.53); *p* = 0.428 G × T; *p* = 0.082
**Parenting Mediators**			
SF: Limit-setting	0.2 (−1.53 to 1.90) *p* = 0.827	0.4 (−1.65 to 2.54) *p* = 0.667	0.3 (−1.02 to 1.65); *p* = 0.639 G × T; *p* = 0.846
SF: Consistency	1.6 (−1.20 to 4.34) *p* = 0.253	0.7 (−1.93 to 3.43) *p* = 0.570	1.2 (−0.72 to 3.04); *p* = 0.220 G × T; *p* = 0.663
**Home Environment Mediators**			
Home Environment Composite Score	−3.7 (−8.01 to 0.66) *p* = 0.093	0.6 (−3.82 to 5.08) *p* = 0.773	−1.5 (−4.56 to 1.51); *p* = 0.319 G × T; *p* = 0.161

T, treatment group; C, control group; CI, confidence interval; FV, fruits and vegetables; SSB, sugar-sweetened beverage; SCS, skin carotenoid scores; SF, structure feeding; G × T, interaction effect between group and time. Bonferroni adjustment conducted to obtain *p* value. * Main effect of time (*p* < 0.01); ** interaction effect (*p* < 0.01).

## Supplementary Materials

The semi-structured focus group interview guide created for both study arms are available online at https://www.mdpi.com/article/10.3390/ijerph18157828/s1.
