# Supplementary material for: Feasibility and Efficacy of the “FUNPALs Playgroup” Intervention to Improve Toddler Dietary and Activity Behaviors: A Pilot Randomized Controlled Trial
Source: Int J Environ Res Public Health. 2021 Jul 23;18(15):7828. doi: 10.3390/ijerph18157828 (PMC8345510; doi:10.3390/ijerph18157828)
Supplement: Supplementary file 1 [file ijerph-18-07828-s001.zip › ijerph-1252892-supplementary.pdf]

Supplementary 1. Third Ward Wellness Study Focus Group Semi-Structured Interview Guide

Background & Context:

The Third Ward Wellness Study for Families is a pilot study of a family based obesity prevention program for toddler age children. The goal is to improve diet, physical activity, sleep, and parenting behaviors through two different approaches. The FUNPALs Playgroup aimed to achieve the behavior change goals through experiential and vicarious learning. The Healthy Toddler Parent Group aimed to achieve the behavior goals through instruction and discussion. The main outcome variables of interest are 1) facilitators and barriers to recruitment, 2) facilitators and barriers of retention and engagement, and 3) authoritative parenting, child diet, child activity, and home environment. Both groups met for 10 weeks on Saturday mornings. We recruited 27 families in the fall and 24 families in the spring and randomly assigned them to each group. One family withdrew. Attendance fluctuated from week to week in both groups.

Objective of Focus Group:

1. The focus group should identify the ways each of the two groups facilitated (or didn't) behavior changes on our outcome variables. This will tell us which program elements are success or less important.
2. A major objective of the focus group is to find out what program elements enhanced engagement and which program elements reduced engagement/retention. Facilitator should spend more time on these questions.

Agenda:

- Welcome and opening remarks.
  - Assign everyone a fake name to use
- Reminders
  - Audiorecording.
  - Refrain from using names.
  - Encourage respect for privacy.
  - Confidentiality limits (i.e., child and incapacitated adult abuse and/or thoughts of harm to self or others)
  - **Discussion questions*…**

ICE BREAKER

1. What was your experience with [*FUN PALs Playgroup*] like?

IDENTIFY FACILITATORS AND BARRIERS TO RETENTION AND ENGAGEMENT

(*Note: For each of the following questions, probe and prompt for families to address the following…*

I. *Interest in topics/activities*
II. *Rapport with facilitators*
III. *Rapport with other families*
IV. *Length of the sessions*

1. What made you sign up for this study?
   a. *Prompt for feelings about their group assignment*
2. What did you like the most about the FUNPALs Playgroup?
3. What was the least favorite part of the FUNPALs Playgroup?

4. What motivated you to attend [*FUNPALs Playgroup*] each week?
5. What barriers did you face getting to the [*FUNPALs Playgroup*] each week? (*Note: those who never came to any sessions should discuss why they did not come at all*).
6. How do you feel about the amount of communication our staff had with you via text, email, phone calls?
7. FUNPALs PLAYGROUP only: To what extent was the program "fun"? What were the program elements that made the playgroup "fun"?
8. How much would you pay for this program? (*without the requirement for data collection pre and post*)

IDENTIFY FACILITATORS AND BARRIERS TO BEHAVIOR CHANGES

9. To what extent did the [*FUN PALs Playgroup*] help you and your child make positive changes in eating habits and nutrition?
    a. What features of [*FUN PALs Playgroup*] helped or didn't help you and your child make positive changes in eating habits and nutrition?
10. To what extent did the [*FUN PALs Playgroup*] help you and your child make positive changes in exercise habits?
    a. What features of [*FUN PALs Playgroup*] helped or didn't help you and your child make positive changes in exercise habits?
11. To what extent did the [*FUN PALs Playgroup*] help you improve your positive parenting skills?
    a. What features of [*FUN PALs Playgroup*] helped or didn't help you and your child make positive changes in positive parenting skills?

Supplementary 2. Third Ward Wellness Study Focus Group Semi-Structured Interview Guide

Background & Context:
The Third Ward Wellness Study for Families is a pilot study of a family based obesity prevention program for toddler age children. The goal is to improve diet, physical activity, sleep, and parenting behaviors through two different approaches. The FUNPALs Playgroup aimed to achieve the behavior change goals through experiential and vicarious learning. The Healthy Toddler Parent Group aimed to achieve the behavior goals through instruction and discussion. The main outcome variables of interest are 1) facilitators and barriers to recruitment, 2) facilitators and barriers of retention and engagement, and 3) authoritative parenting, child diet, child activity, and home environment. Both groups met for 10 weeks on Saturday mornings. We recruited 27 families in the fall and 24 families in the spring and randomly assigned them to each group. One family withdrew. Attendance fluctuated from week to week in both groups.

Objective of Focus Group:

3. The focus group should identify the ways each of the two groups facilitated (or didn't) behavior changes on our outcome variables. This will tell us which program elements are success or less important.
4. A major objective of the focus group is to find out what program elements enhanced engagement and which program elements reduced engagement/retention. Facilitator should spend more time on these questions.

Agenda:

- Welcome and opening remarks.
  - Assign everyone a fake name to use
- Reminders
  - Audiorecording.
  - Refrain from using names.
  - Encourage respect for privacy.
  - Confidentiality limits (i.e., child and incapacitated adult abuse and/or thoughts of harm to self or others)
  - **Discussion questions*…**

ICE BREAKER

2. What was your experience with [*Healthy Families Parent Class*] like?

IDENTIFY FACILITATORS AND BARRIERS TO RETENTION AND ENGAGEMENT

(*Note: For each of the following questions, probe and prompt for families to address the following…*

V. Interest in topics/activities
VI. Rapport with facilitators
VII. Rapport with other families
VIII. Length of the sessions

12. What made you sign up for this study?
    a. *Prompt for feelings about their group assignment*
13. What did you like the most about the Healthy Toddler Parent Group?
14. What was the least favorite part of the Healthy Toddler Parent Group?

15. What motivated you to attend [*Healthy Toddler Parent Class*] each week?
16. What barriers did you face getting to the [*Healthy Toddler Parent Class*] each week? (*Note: those who never came to any sessions should discuss why they did not come at all*).
17. How do you feel about the amount of communication our staff had with you via text, email, phone calls?
18. HTPG families only: What were the barriers and facilitators to using the Child Watch Center?
19. How much would you pay for this program? (*without the requirement for data collection pre and post*)

IDENTIFY FACILITATORS AND BARRIERS TO BEHAVIOR CHANGES

20. To what extent did the [*Healthy Toddlers Parent Class*] help you and your child make positive changes in eating habits and nutrition?
    a. What features of [*Healthy Toddlers Parent Class*] helped or didn't help you and your child make positive changes in eating habits and nutrition?
21. To what extent did the [*Healthy Toddlers Parent Class*] help you and your child make positive changes in exercise habits?
    a. What features of [*Healthy Toddlers Parent Class*] helped or didn't help you and your child make positive changes in exercise habits?
22. To what extent did the [*Healthy Toddlers Parent Class*] help you improve your positive parenting skills?
    a. What features of [*Healthy Toddlers Parent Class*] helped or didn't help you and your child make positive changes in positive parenting skills?
